# Synthesis and molecular docking study of novel COVID-19 inhibitors

**DOI:** 10.3906/kim-2012-55

**Published:** 2021-06-30

**Authors:** Zuhal GERÇEK, Deniz CEYHAN, Erol ERÇAĞ

**Affiliations:** 1 Department of Chemistry, Faculty of Arts and Sciences, Bülent Ecevit University, Zonguldak Turkey; 2 Department of Chemistry, Faculty of Art and Science, Tekirdağ Namık Kemal University, Tekirdağ Turkey

**Keywords:** COVID-19, SARS-CoV-2, molecular docking study, antiviral drug

## Abstract

In 2020, the world tried to combat the corona virus (COVID-19) pandemic. A proven treatment method specific to Severe Acute Respiratory Syndrome Coronavirus-2 (SARS-CoV-2) is still not found. In this study, seven new antiviral compounds were designed for COVID-19 treatment. The ability of these compounds to inhibit COVID-19’s RNA processing was calculated by the molecular docking study. It has been observed that the compounds can have high binding affinities especially against NSP12 (between -9.06 and -8.00 kcal/mol). The molecular dynamics simulation of NSP12-ZG 7 complex proved the stability of interaction. The synthesis of two most active molecules was performed by one-pot reaction and characterized by FT-IR, ^1^H-NMR, ^13^C-NMR, and mass spectroscopy. The compounds presented with their synthesis are inhibitory core structures against SARS-CoV-2 infection.

## 1. Introduction

The most interested subject in 2020 is corona virus disease, which was named as COVID-19 by the WHO (World Health Organization) on the February 11, 2020 [1], This novel coronavirus is called as Severe Acute Respiratory Syndrome Coronavirus 2 (SARS-CoV-2) by the international virus classification commission. Viruses in the corona family cause diseases in respiratory, gastrointestinal, hepatic, and central nervous system in both humans and animals [2]. Due to the respiratory transmission of SARS-CoV-2 from person to person, it has led to the formation of pandemic conditions in a short time. The world has become familiar with corona virus first with SARS (Severe Acute Respiratory Syndrome) epidemic, and then with the MERS (Middle East Respiratory Syndrome) epidemic [3]. The cause of pneumonia in COVID-19 cases is revealed as unique b-CoV strain [4]. The scientific world does not have an approved treatment specific to SARS-CoV-2. Luckily, it was shown that the novel b-CoV shows 88% similarity to the (SARS)-like coronaviruses, and about 50% to the MERS CoV. Therefore, drugs used for SARS and MERS have come forth again [5]. 

There are many potential drug candidates for the treatment of COVID-19 such as, oseltamivir [2], lopinavir/ ritonavir [6, 7], nucleoside analogues and nucleotide inhibitors [8] remdesivir [6, 9], tenofovir, ribavirin, sofosbuvir, galidesivir [10] antibiotics [11] and chloroquine and hydroxychloroquine [12,13]. Alternatively, various phytochemicals were used against SARS-CoV-2 virus too. Examples are, belachinal, macaflavanone E and vibsanol B [14], flavone and coumarine derivatives [15], saikosaponins [16], crocin, digitoxigenin, and ß-eudesmol [17], d-viniferin, myricitrin, Taiwan homoflavone A, lactucopicrin 15-oxalate, nymfolide A, afzelin, biorobin, hesperidin and phyllaemblicin B [18], and theophylline derivatives [19].

Hydroxychloroquine, which is mainly used for the treatment of malaria [20] was the first drug to be considered suitable for use in the treatment of COVID-19. On 17 June 2020, WHO announced that the research examining the effects of hydroxychloroquine in the treatment of COVID- 19 was cancelled. It has been reported that the drug does not have a positive effect on the mortality rate and duration of hospital stay compared to standard treatments [21]. On July 1, 2020 FDA (Food and Drug Administration) issued a warning stating that hydroxychloroquine causes heart rhythm problems, blood and lymph system disorders, kidney injuries, and liver problems and failure [22].

Remdesivir is an antiviral drug that block viral RNA synthesis of RNA viruses such as SARS and MERS. The antiviral efficacy of the drug has been proven in many in vitro studies [23, 24]. However, it has not been approved for the COVID- 19 treatment yet. As the emergency situation continues, FDA has issued an authorization on the use of the drug on hospitalized patients receiving COVID-19 treatment [22].

Since there are limited number of antiviral drugs proven to be effective in COVID-19 treatment, there is an urgent and pressing need to design and synthesize of new antiviral drugs specific to COVID-19. 

Nonstructural proteins (NSPs) have been reported as effective targets for small molecular drugs in SARS and MERS cases [25]. In this study, we have studied potentials of novel compounds that attack to the Mpro, NSP12, NSP15, and NSP16 regions of SARS-CoV-2. 

Ongoing studies have shown that the main protease of coronavirus (Mpro) is responsible for virus replication. Molecules capable of inhibiting the main protease will potentially prevent the COVID-19 virus from the reproduction itself inside the cell. Hence, main protease of coronavirus (Mpro) has become the target of new anti-corona virus drugs [26-28]. 

A total of 16 non-structural proteins (NSPs) of corona virus involved in genome replication and transcription were clearly identified [29]. All NSPs have their own functions. Among them, NSP12 has an RNA polymerase activity [30]. NSP15 is the most popular target of antiviral drugs due to its endonuclease activity. Function of NSP16 is to mediate the 5’ methylation of virus genome [30].

**Figure 1:**
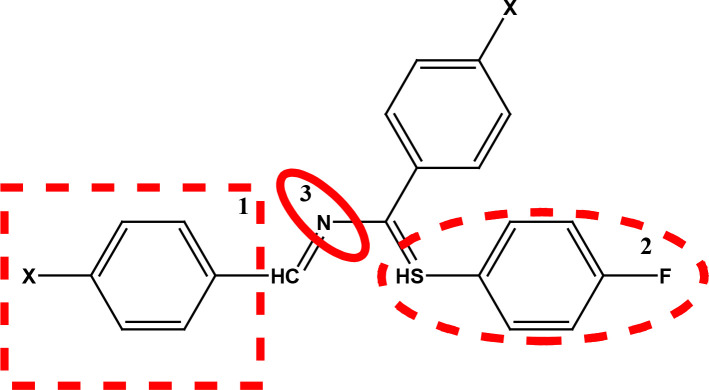
The skeleton of novel compounds.

After examining the current literature, the main skeleton of the novel COVID-19 drugs was determined (Figure 1). Seven new compounds are proposed with different aryl groups (Table 1). 

**Table 1:** Structures of proposed molecules.

Compound	Structure
ZG-1	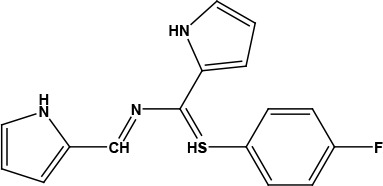
ZG-2	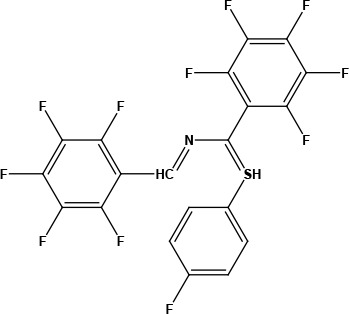
ZG-3	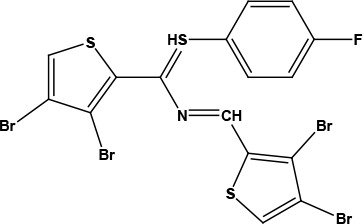
ZG-4	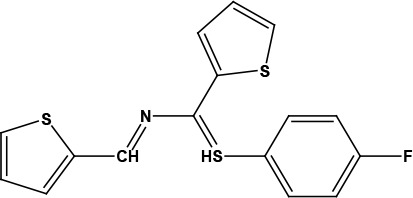
ZG-5*	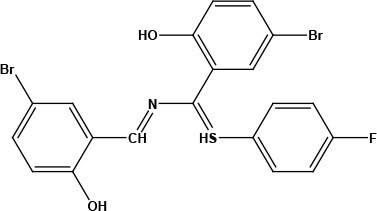
ZG-6	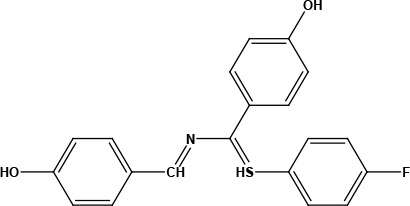
ZG-7*	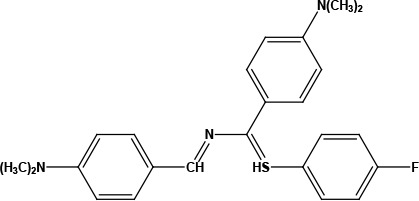

* Synthesized compounds.

The new drug candidate compounds consist of 3 key constituents: various substituted aromatic ring (1), p-floro thiophene (2) and a nitrogen (3). 

The first part (1) can be changed from substituted benzene to hetero aromatic rings. Also, substituents on the aromatic ring can be changed easily by using different aldehydes. The sulphur in the second component was included in the structure due to its capacity to make H-bonding. The fluorine in the ring is at the para position due to the fact that fluorinated compounds in the para position are found the most active derivative in the literature [31]. Nitrogen atom is the main poi chemicals onto the targeted segments of protein of interest in COVID-19 was used as to determine the binding energies. Previously, a detailed study on the well-known antiviral hydroxychloroquine and remdesivir drugs was done as a validation methodology.

**Figure 2:**
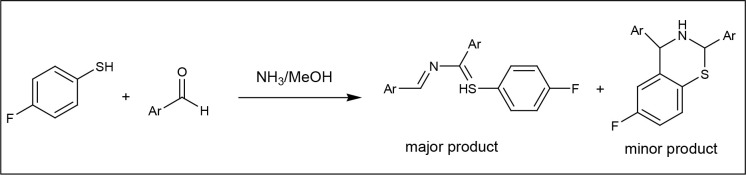
General reaction route.,

Molecules with the highest scores in docking studies (ZG-5 and ZG-7) have been synthesized and characterized (Figure 2). 

## 2. Materials and methods

### 2.1. Molecular docking studies

Molecular docking studies were performed to provide a theoretical perspective for possible molecular interactions of ZG-series compounds and reference molecules with the target proteins. The theoretical binding affinities were determined by energy minimization from docking calculation results. Molecular docking calculations, energy minimization, and molecular visualization of docking results were carried out by using the Molecular Operating Environment software package (MOE, v2019.0102, Chemical Computing Group ULC). Preparation of ZG-series (ZG 1-7) and model inhibitor molecules for molecular docking was performed with MarvinSketch software. Before the docking process, the drawing and editing of the novel ZG-series compounds in SD File format was done with the MarvinSketch suit program. These molecular structures have been protonated, added charges, and conformation minimization was performed with the root mean square gradient (RMS 0.001 kcal/mol/A2) by using the MMFF94 Forcefield parameters, which can be accessed in Energy Minimization protocols of these software [32,33]. 

Docking studies of ZG-series compounds were carried out for four different important target structures named 3CLpro, NSP12, NSP15, and NSP16 from COVID-19. The X-ray crystal structures as three-dimensional coordinates of these target proteins was obtained from the Research Collaboratory for Structural Bioinformatics (RCSB) Protein Data Bank [34]. For use in docking calculations, structures with PDB IDs of 7BQY [35] for 3CLpro, 7BV2 [36] for NSP12, 6WXC [37] for NSP15 and 6WKQ [34] for NSP16 were chosen as crystal structure models corresponding to these target proteins. Structural defects in these target proteins were eliminated automatically with the “structure preparation” module of MOE suit software. Default parameters of MOE Protonate 3D module were used to determine and optimize the overall low potential energy configuration of the terminal amides, hydroxyls, thiols, histidines, and hydrogenation positions of the titratable groups in a certain pH value throughout the system (Temperature 300 Kelvin, pH 7, solvent 0.1 M, electrostatic energy cutoff 15 A with Generalized Born Model_GB/VI; van der Waals 800R3 cutoff 10 A, MOE Protonate 3D) [38]. The energy minimization of the system was performed with the Amber12: EHT Forcefield parameters, root mean square deviation (RMSD) with the gradient of 0.001 kcal/mol/A2, which can be reached in MOE energy minimization protocols [39,40]. Possible ligand binding sites in the minimized protein were determined by MOE “Site Finder” module. The Site Finder methodology is a geometric method based on Alpha Shapes, a generalization of convex surfaces developed by Edelsbrunner [41].

Local docking of ZG-series compounds and model inhibitors to the active site of these targets proteins was performed via MOE using the default docking calculation parameters. The average score of the top 10 final docking poses defined by the binding minimum energy (kcal/mol) for each compound was used as the final molecular docking score results. London dG scoring function was used for docking calculations. The London dG scoring function estimates the free energy of binding the ligand at a particular pose in a target structure. This scoring function is explained in detail in the user manual of the MOE software. After the initial scoring function for the obtained docking poses, the GBVI/WSA ΔG scoring function was used as the final docking scoring methodology. The GBVI/WSA dG is a forcefield-based scoring function, which estimates the free energy of binding of the ligand from a given pose [42]. This scoring function is explained in detail in the user manual of the MOE software. 

### 2.2. Molecular dynamics simulation 

In order to prove the stability of the NSP12-ZG 7 interaction, a short molecular dynamics simulation was performed. NAMD (v2.9) program was used for molecular dynamics simulation and VMD programs were used for analysis of simulation results [43,44]. Minimization process was performed for 100 pico-second (ps) in an aqueous medium in the presence of Na^+^ and Cl^-^ ions at the appropriate concentration for NSP12 protein and NSP12-ZG 7 complex. After that the minimized structures were heated to 36 °C under 1 atm pressure for 100 ps. Then, the simulation process was carried out in an aqueous environment at 36 °C and 1 atm pressure for 2000 ps. 

### 2.2. Chemistry

All reagents and solvents were of commercial origin and used without further purification. ^1^H- NMR and ^13^C-NMR spectra were recorded with a Bruker Ultra Shield Plus ultra-long-hold-time spectrometer with DMSO-d_6_ as the solvent. All chemical shifts are given relative to tetramethylsilane. FT-IR spectra were recorded with Perkin Elmer Spectrum 100 (PerkinElmer, Inc., Waltham, MA, USA). Mass data were obtained by Water Xevo TQD system. 

The general procedure was as follows. The novel compounds were synthesized by one-pot reaction modified from Piste et.al. [45]. 4-florobenzenethiol (10 mmol) in 10 ml methanol was added in aromatic aldehyde (20 mmol) and 10 ml 30% ammonia solution. The reaction mixture was stirred at room temperature till crystalline product separated out (3 days). The solid was filtered and purified by recrystallization with methanol.

The Synthesis of (E/Z) - (4-fluorophenylthio) -N-(5-bromo-2-hydroxybenzylidene) (5-bromo-2-hydroxyphenyl) methanamine, ZG-5: Yellow solid, mp: 98-100°C.

IR(KBr)n(cm^-1^): 3500-3200 (OH); 3000 (C-H); 1650 (C=); 1600 (C=N); 1400-1000 (C-F); 650 (C-S). ^1^H NMR (DMSO-d_6_, 600 MHz) d (ppm): ^1^H NMR (DMSO-d_6_, 600 MHz) d (ppm): 11.05 (broad s, 2H); 10.19 (s, 1H); 7.68 (d, 1H, *J* = 2.5 Hz); 7.63 (d, 1H, *J* = 2.5 Hz); 7.61 (d, 1H, *J* = 8.8 Hz); 7.54-7.52 (m, 2H); 7.23 (t, 2H, *J* = 8.8 Hz); 6.96 (d, 2H, *J* = 8.8 Hz). ^13^C NMR (DMSO-d_6_, 150 MHz) d (ppm): 190.02, 163.19, 161.57, 160.29, 138.91, 131.27, 130.82, 124.43, 120.33, 117.13, 116.99, 111.14. m/z: M^+^: 512.55 (molecular weight: 511.2).

The synthesis of (E/Z) -4- ((4-(dimethylamino) benzylideneamino) (4-fluorophenylthio)methyl) -N,N-dimethylbenzenamine, ZG-7: grey solid, mp: 74-76°C.

IR(KBr)n(cm^-1^): 2980-2700 (C-H); 1670 (C=C); 1700 (C=N); 1130 (C-F); 650 (C-S). ^1^H NMR (DMSO-d6, 600 MHz) d (ppm): 9.65 (s, 1H), 7.66 (d, 4H, *J* = 8.8 Hz), 7.54 (m, 2H), 7.23 (t, 2H, *J* = 6.9 Hz), 6.76 (d, 4H, *J* = 8.8 Hz), 3.01 (s, 12 H). ^13^C NMR (DMSO-d_6_, 150 MHz) d (ppm): 190.29, 163.20, 161.57, 154.60, 131.26, 131.21, 124.93, 117.13, 116.99, 111.48, 40.07. m/z: M^+^: 409.03 (molecular weight: 407.55).

## 3. Results 

### 3.1. Chemistry

Presented molecules were synthesized by one-pot reaction modified from Piste et.al. [45].

The purification of crude products was performed by recrystallization with methanol. All characterization of molecules was done by FT-IR, ^1^H NMR, ^13^C NMR, and mass spectroscopy. ^1^H NMR spectrum ZG-5 contains a broad singlet at 11.05 ppm belongs to –OH, a singlet at 10.19 ppm for imine (–HC=N-) proton, signals belong to aromatic parts appear between 7.69-6.96 ppm. In ^13^C NMR spectrum signals at 190.02 ppm for -CS- carbon and at 160.29 ppm for imine carbon confirm the structure. Also, mass spectrum m/z: M^+^: 512.55 (molecular weight: 511.2) proofs the structure. Signal of imine proton appears at 9.65 ppm, signals of aromatic rings can be seen between 7.67-6.76 ppm and CH_3_ signal can be seen as singlet at 3.01 ppm in ^1^H NMR spectrum of ZG-7. Signal belongs to -CS- carbon can be seen at 190.29 ppm, signal of imine carbon present at 163.20 ppm in ^13^C NMR spectrum of ZG-7. In mass spectrum, m/z: M^+^: 409.03 (molecular weight: 407.55) confirmed the structure.

FT-IR spectra contain typical C-F (1400-1000 cm^-1^), C-S (650 cm^-1^) and C=N (1600 cm^-1^) signals. 

### 3.2. Molecular docking studies 

**Figure 3:**
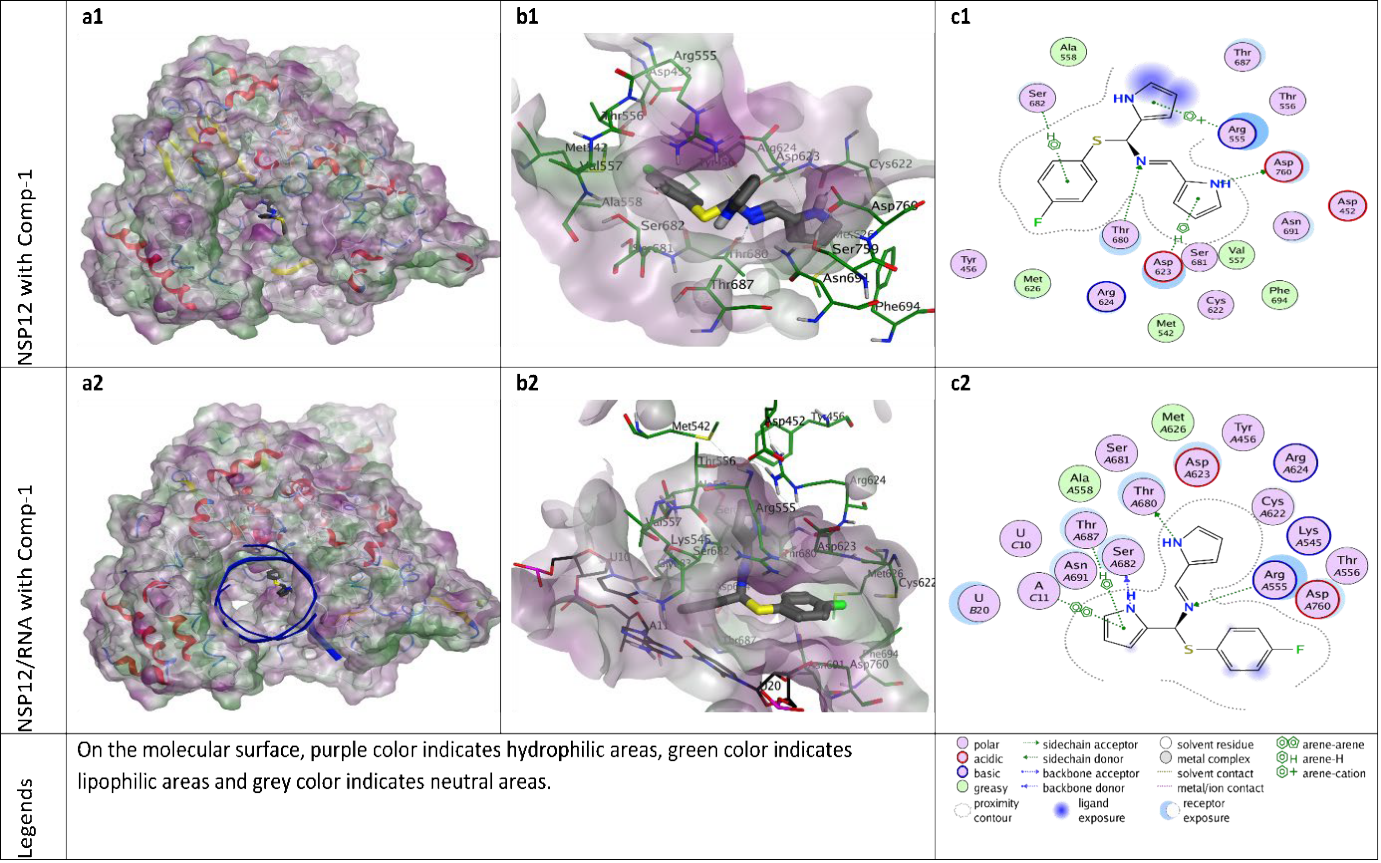
Docking results of ZG-1 with NSP12 and NSP12-RNA complex. Figures a1 and a2 show the localization of ZG-1 on the NSP12 and NSP12-RNA complex presented with the lipophilicity surface area, respectively. b1, b2 show 3D interactions and c1, c2 show 2D interactions of ZG-1 with NSP12 and NSP12-RNA complex, respectively.

**Figure 4:**
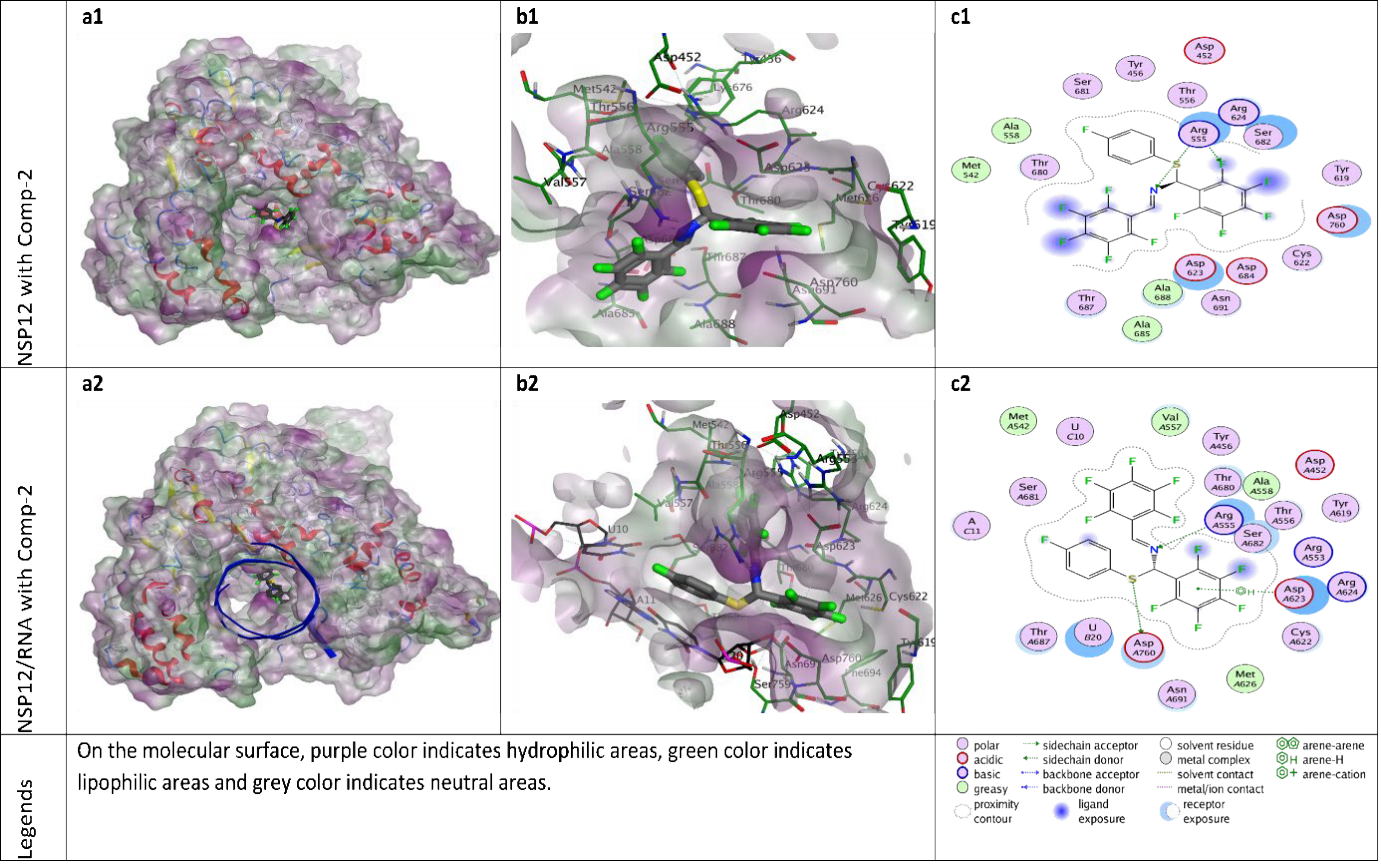
Docking results of ZG-2 with NSP12 and NSP12-RNA complex. Figures a1 and a2 show the localization of ZG-2 on the NSP12 and NSP12-RNA complex presented with the lipophilicity surface area, respectively. b1, b2 show 3D interactions and c1, c2 show 2D interactions of ZG-2 with NSP12 and NSP12-RNA complex, respectively.

**Figure 5:**
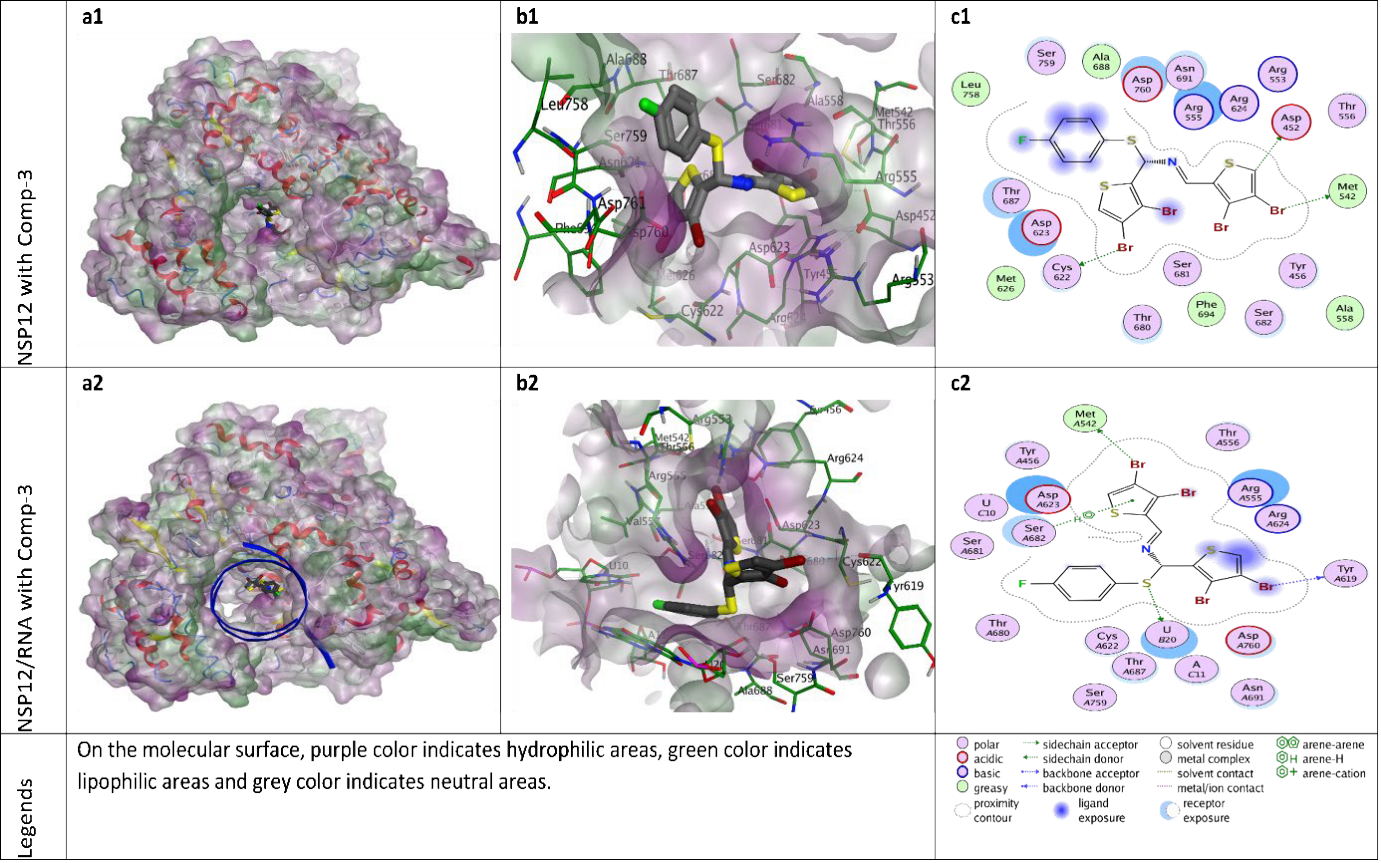
Docking results of ZG-3 with NSP12 and NSP12-RNA complex. Figures a1 and a2 show the localization of ZG-3 on the NSP12 and NSP12-RNA complex presented with the lipophilicity surface area, respectively. b1, b2 show 3D interactions and c1, c2 show 2D interactions of ZG-3 with NSP12 and NSP12-RNA complex, respectively.

**Figure 6:**
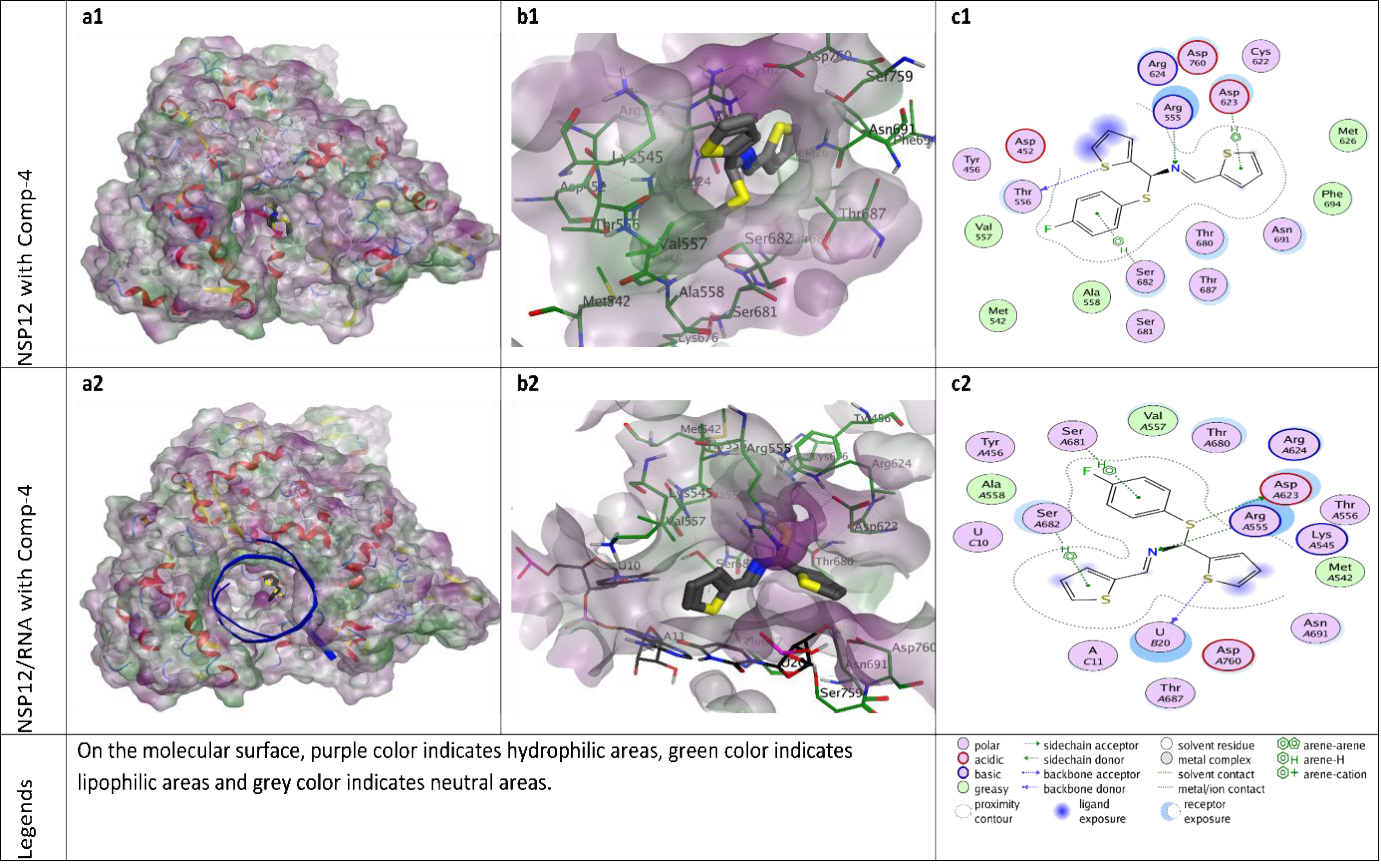
Docking results of ZG-4 with NSP12 and NSP12-RNA complex. Figures a1 and a2 show the localization of ZG-4 on the NSP12 and NSP12-RNA complex presented with the lipophilicity surface area, respectively. b1, b2 show 3D interactions and c1, c2 show 2D interactions of ZG-4 with NSP12 and NSP12-RNA complex, respectively.

**Figure 7:**
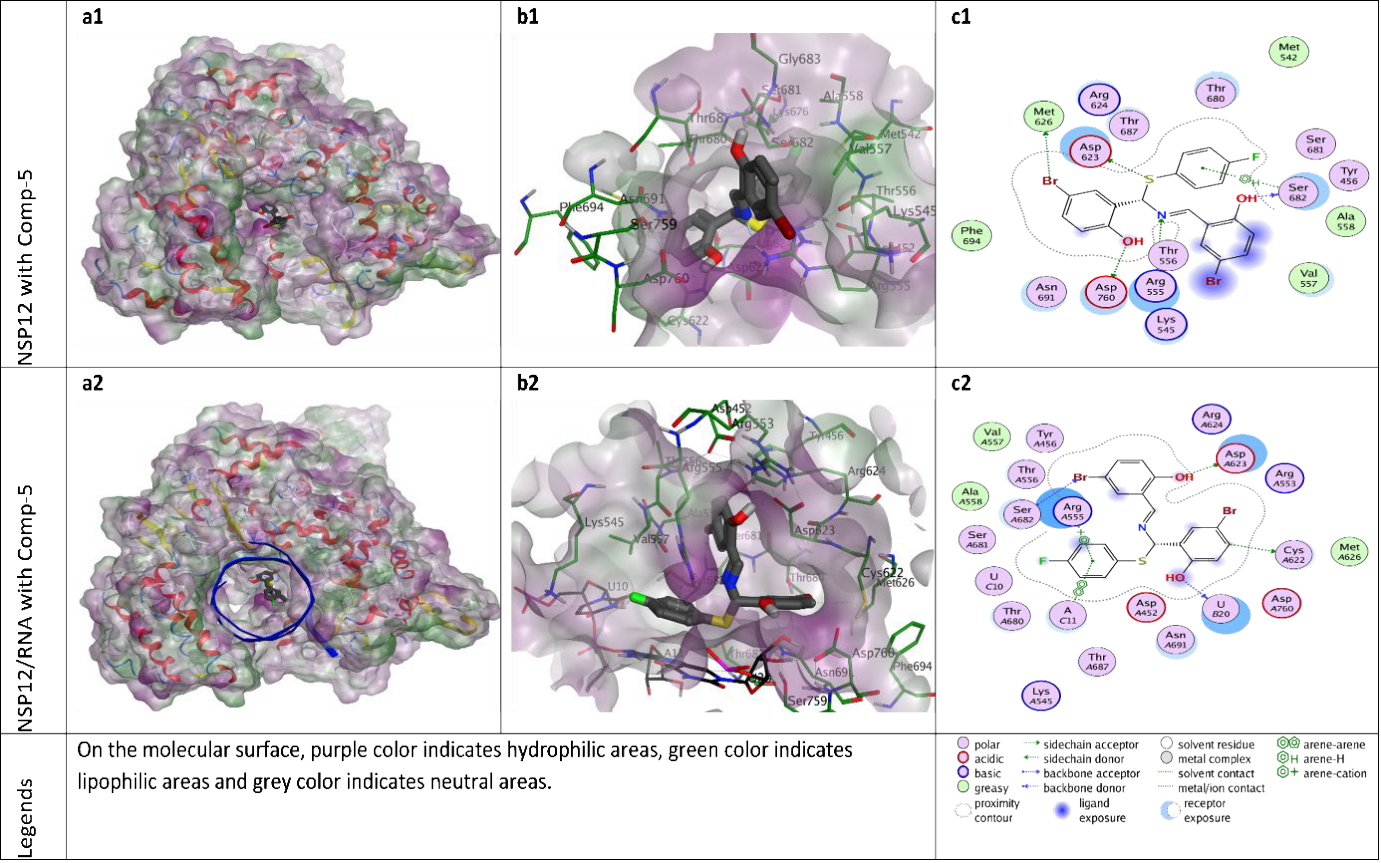
Docking results of ZG-5 with NSP12 and NSP12-RNA complex. Figures a1 and a2 show the localization of ZG-5 on the NSP12 and NSP12-RNA complex presented with the lipophilicity surface area, respectively. b1, b2 show 3D interactions and c1, c2 show 2D interactions of ZG-5 with NSP12 and NSP12-RNA complex, respectively.

**Figure 8:**
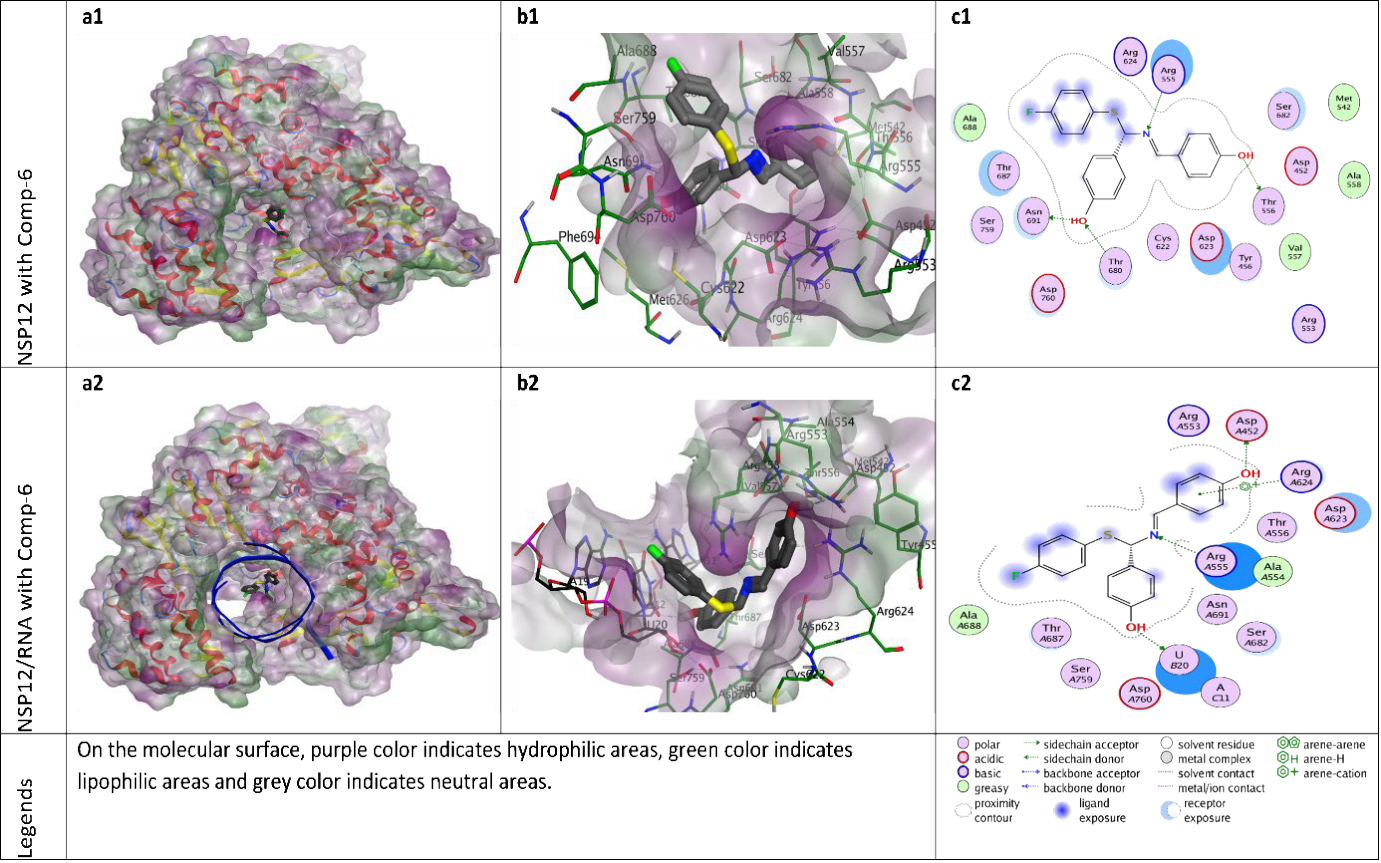
Docking results of ZG-6 with NSP12 and NSP12-RNA complex. Figures a1 and a2 show the localization of ZG-6 on the NSP12 and NSP12-RNA complex presented with the lipophilicity surface area, respectively. b1, b2 show 3D interactions and c1, c2 show 2D interactions of ZG-6 with NSP12 and NSP12-RNA complex, respectively.

**Figure 9:**
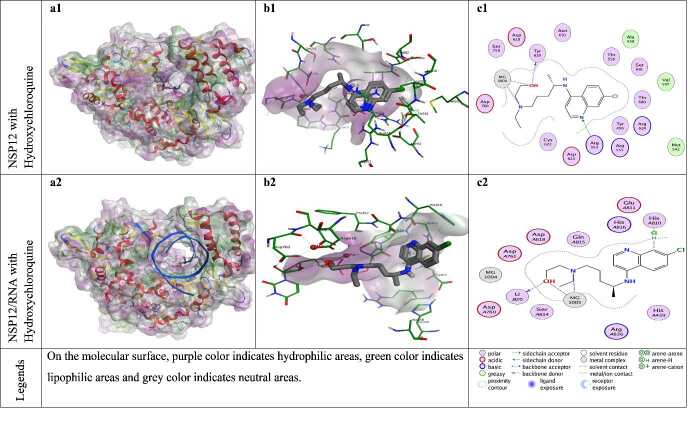
Docking results of ZG-7 with NSP12 and NSP12-RNA complex. Figures a1 and a2 show the localization of ZG-7 on the NSP12 and NSP12-RNA complex presented with the lipophilicity surface area, respectively. b1, b2 show 3D interactions and c1, c2 show 2D interactions of ZG-7 with NSP12 and NSP12-RNA complex, respectively.

**Figure 10:**
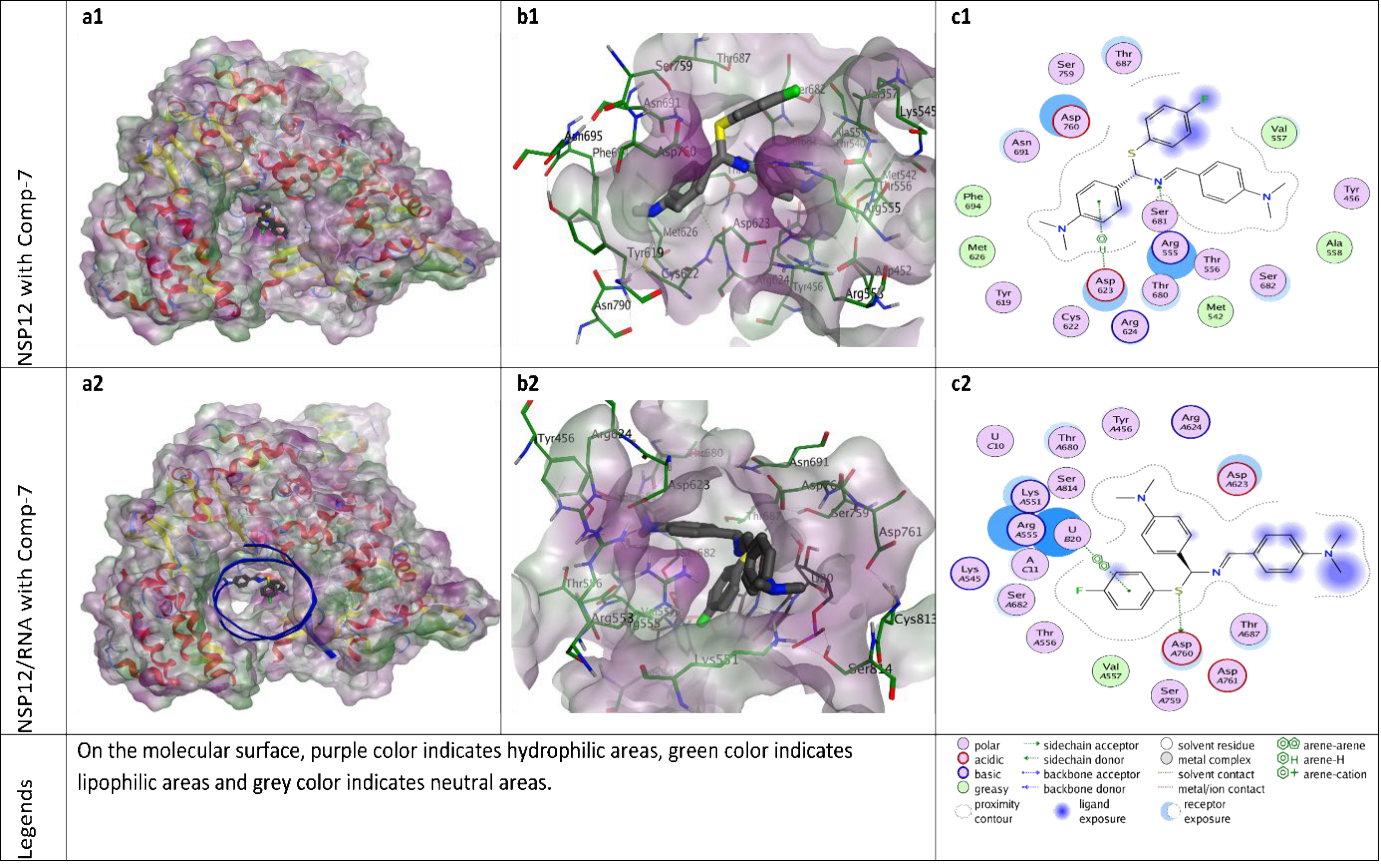
Docking results of hydroxychloroquine with NSP12 and NSP12-RNA complex. Figures a1 and a2 show the localization of hydroxychloroquine on the NSP12 and NSP12-RNA complex presented with the lipophilicity surface area, respectively. b1, b2 show 3D interactions and c1, c2 show 2D interactions of hydroxychloroquine with NSP12 and NSP12-RNA complex, respectively.

**Figure 11:**
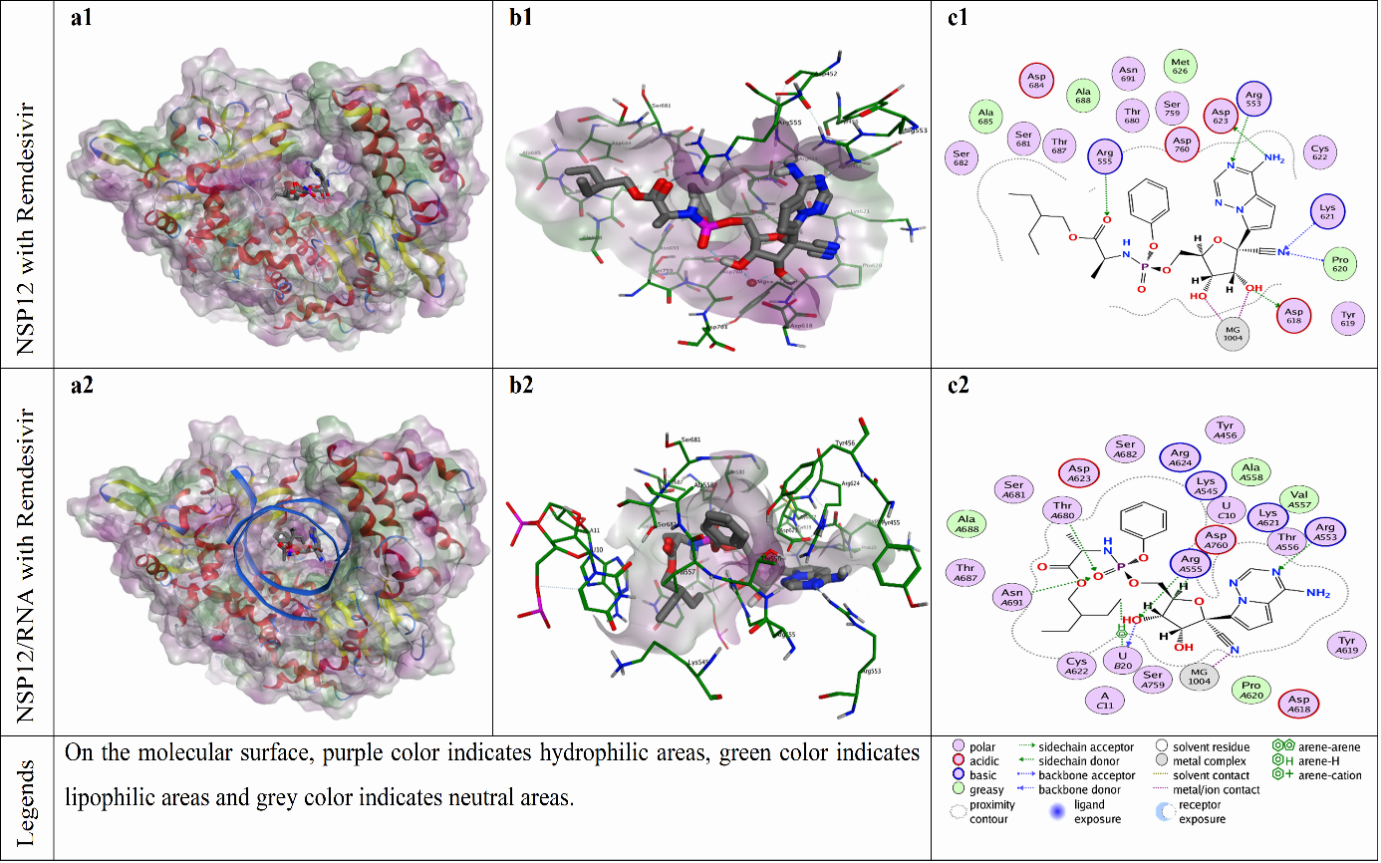
Docking results of remdesivir with NSP12 and NSP12-RNA complex. Figures a1 and a2 show the localization of remdesivir on the NSP12 and NSP12-RNA complex presented with the lipophilicity surface area, respectively. b1, b2 show 3D interactions and c1, c2 show 2D interactions of remdesivir with NSP12 and NSP12-RNA complex, respectively.

Docking analyses were performed to understand the molecular interaction mechanisms between ZG-series compounds and the target proteins. In addition of these, docking analyses of model inhibitor molecules hydroxychloroquine and remdesivir, reported to be effective against COVID-19, were also performed. Docking results of the all compounds are given in Table 2 and Figure 3-11. 

**Table 2:** Docking scores (kcal/mol) of ZG compounds and reference inhibitor molecules with target proteins.

Compound	NSP5 (Mpro)	NSP12	NSP12/RNA	NSP15	NSP16
ZG-1	-6.29-7.28*	-6.38-7.50*	-6.65-8.00*	-6.45-7.24*	-6.43-7.05*
ZG-2	-6.77-7.96*	-6.80-8.16*	-7.02-8.24*	-6.72-7.34*	-6.51-7.36*
ZG-3	-7.19-7.89*	-7.03-8.13*	-7.01-8.46*	-7.18-7.38*	-6.81-7.34*
ZG-4	-6.54-7.02*	-6.28-7.69*	-6.79-8.01*	-6.71-6.86*	-6.26-6.95*
ZG-5	-7.22-7.54*	-7.29-8.60*	-7.72-9.06*	-7.21-7.60*	-7.05-7.89*
ZG-6	-6.93-7.56*	-6.75-7.77*	-7.03-8.15*	-6.87-7.45*	-6.62-7.25*
ZG-7	-7.46-8.79*	-7.74-8.90*	-7.72-8.99*	-7.52-7.80*	-7.09-7.43*
hydroxy- chloroquine	-6.97 -7.31*	-7.59 -8.19*	-7.92 -8.69*	-6.18 -6.68*	-6.89 -7.59*
remdesivir	-8.73 -9.62*	-9.06 -10.70*	-9.51 -12.01*	-7.36 -7.84*	-8.65 -10.15*

*Energy minimization value (kcal/mol).

According to the docking results, ZG-series compounds showed a higher activity with NSP12-RNA complex protein than other target proteins. The ZG-series compounds with the highest docking score are ZG-3, ZG-5, and ZG-7. These compounds are able to show binding affinity to NSP12 protein with an energetic value of -8.13, -8.60 and -8.90 kcal/mol, respectively. The binding affinities of these compounds to NSP12-RNA complex are -8.46, -9.06 and -8.99 kcal/mol, respectively. The reference molecules hydroxychloroquine and remdesivir showed binding affinity at -7.59 and -9.06 kcal/mol for the NSP12 protein, while -7.92 and -9.51 kcal/mol for the NSP12-RNA complex. 

The catalytic site major residues of NSP12 that interact with ZG-series compounds are Asp452, Met542, Arg553, Arg555, Thr556, Cys622, Asp623, Arg624, Met626, Thr680, Ser682, Asn691, Phe694, Ser759, Asp760. NSP12 (PDB ID: 7BV2) has magnesium ions in its catalytic domain. Since magnesium ions take part in natural catalytic processes, they are preserved in docking calculation and considered as a catalytic site component (as residues MG1004 and MG1005). It was seen in docking calculation that these magnesium ions, especially MG1004, form metal ligation with all ZG-series compounds. However, no interaction was observed between these magnesium ions and model compounds in the catalytic domain. 

When the different docking poses were examined together, it was observed that the NSP12 residues that interact most frequently with ZG-series compounds are Arg555, Asp623, and Ser682. Arg555 residue generally shows strong side-chain hydrogen bond (acceptor) interactions with ZG-series compounds except ZG-1. Residues Asp623 and Ser682 usually show arene attraction (pi- interactions) in different docking poses of ZG-series compounds (Figures 3-11). 

The residues of the NSP12 protein that show the highest interaction with model compounds (hydroxychloroquine and remdesivir) are Arg553 and Arg555. These residues usually show strong sidechain hydrogen bond (acceptor) interaction in different docking poses of model (reference) compounds. Arg553 residue shows side chain hydrogen bond (acceptor) interactions with hydroxychloroquine and remdesivir with the different docking poses. Arg555 residue had strong side chain hydrogen bond (acceptor) interactions with model compound remdesivir, strong arene attraction interaction with model compound hydroxychloroquine in only one docking pose. 

NSP12-RNA complex residues that frequently interact with ZG-series compounds are Arg555, Ser682, pU20 (primer Uracil nucleotide) and tU10 (template -Uracil nucleotide). Arg555 residue shows side chain hydrogen bond (acceptor) interactions with ZG-series compounds, except for ZG-6 at different docking poses. Ser682 residue shows arene attraction interactions with ZG-series compounds, except for ZG-2 and ZG-7. Ser682 residue also shows backbone hydrogen bond (donor) interactions with ZG-1, ZG-4 and ZG-5. 

Strong metal ligation interactions are generally observed between the magnesium ions (especially the magnesium ion called MG1004 residue in 7BV2.PDB protein model) and the reference compounds hydroxychloroquine and remdesivir, which are the natural components of NSP12 or NSP12-RNA complex. However, magnesium ions showed no interaction with the ZG-series compounds. 

One of the common features of ZG-series compounds is that they contain “S” and “N” elements together. These elements are important in the interaction of ZG-series compounds with the protein NSP12 (with or without RNA component). The “N” or “NH” group indicates the H-bond interactions with the 7BV2 protein model (NSP12-RNA) in all compounds except ZG-3. The S-elements in the ZG-series compounds shows strong H-bond interactions with 7BV2 protein model (NSP12-RNA) residues, except for ZG-1 and ZG-6. 

The N-element or NH-group in ZG-1 is able to establish H-bond interactions with Thr680 and Asp760 residues of NSP12 (7BV2.PDB), and H-bond interactions with key residues of NSP12 such as Arg555, Ser682 and Thr680 in the presence of the RNA components. However, it seems that P-P, P-H and P-cation interactions are important in stabilizing ZG-1 on this protein (Figure 3). 

Van der Waals polar and hydrophobic interactions of the F-element appear to be important for the binding of ZG-2 to NSP12. It was also observed that F-element in ZG-2 can form H-bond with Arg555 residue of NSP12 protein. In addition, the S-element of ZG-2 appears to form H-bond and acidic interactions with Asp760 (Figure 4). 

ZG-3 is able to establish H-bond interactions through the Br atoms with residues Met542, Tyr619 and Cys622 of the 7BV2 PDB model (NSP12-RNA). The linker S element of ZG-3 forms strong H-bond with the uracil nucleotide (pU-B20) of the primary RNA component in the 7BV’ PDB model (Figure 5). 

The N of ZG-4 established H-bond interaction with the Arg555 residue of NSP12 and the S element in the ring structure with the Thr556 residue. In the presence of RNA, the cyclic S element establishes a significant H-bond interaction with primary Uracil (pU-B20), while the linker S element performs an H-bond interaction with the Asp623 residue of NSP12. Pi-H interactions, including aromatic structures, are also important in the stabilization of ZG-4 on the protein (Figure 6). 

ZG-5 binds to the NSP12 protein via a large number of H-bonds. The compound ZG-5 establishes H-bond interactions with the residues Thr556, Asp623, Met682, and Asp760 of NSP12 through the N, S, Br elements, OH (1) and OH (2) functional groups, respectively. When the RNA components in the 7BV2.PDB model are included in the docking calculations, ZG-5 shows Van der Waals interaction with template Uracil nucleotide (tU10), pi-pi interaction with template Adenine nucleotide (tA11), and H-bond interactions with primary Uracil nucleotide (pU20) (Figure 7). 

In the interactions of the ZG-6 with the Apo-NSP12 protein, the H-bonding potential of the functional OH-groups in the compound is important. In the presence of RNA molecules, the ZG-6 establishes the Van der Waals interaction with template-Adenine nucleotide (tA11) and H-bond interaction with the primary-Uracil nucleotide (pU20) through its OH-group (Figure 8). 

Compound ZG-7 revealed hydrogen bond with Arg555, hydrophobic interaction with Met542, Val557, Ala558, Met626 and Phe694; a Pi-H interaction with Asp623; Van der Waals interaction with Tyr456, Arg555, Thr556, Tyr619, Cys622, Asp623, Arg624, Thr680, Ser681, Ser682, Thr687, Asn691, Ser759, Asp760 and Ser814; acidic interaction with Asp623, Asp760 and Asp761; basic interaction with Arg555 and Arg624 or Lys551, Lys545 residues of NSP12 (with or without RNA molecules). In the presence of RNA components, one H-bond is formed between the S-element of ZG-7 and the Asp760 residue of NSP12 protein. At the same time, ZG-7 shows Van der Waals interaction with template RNA nucleotides U10 and A11, pi-pi interaction with primer RNA nucleotide U20 (Figure 9). 

The reference compound hydroxychloroquine establishes H-bond interaction with residues Arg553 and Tyr619 of NSP12 protein. While there are RNA components, hydroxychloroquine establishes H-bond interaction with pU20 (primer Uracil-B20). H-bonds are formed between the reference molecule remdesivir and residues Arg553, Arg555, Asp618, Pro620, Lys621, Asp623, Thr680, Asn691 and Asp760 of NSP12 protein (with or without RNA). Similarly, remdesivir can establish H-bond interaction with pU20 (primer Uracil B20). Reference molecules (hydroxychloroquine and remdesivir) are able to establish metalic and ionic interactions with magnesium ions in the NSP12 protein, unlike ZG-series compounds (1-7). 

### 3.3. Molecular dynamics simulation 

The interactions between NSP12 and ZG 7 in different time frames according to molecular dynamics simulation are given in Figure 12. The NSP12-ZG 7 interaction at 2000 ps in the molecular dynamics simulation is given in Figure 13. Root-mean-square deviation (RMSD) plots of NSP12 and NSP12-ZG 7 complex throughout the simulation are given in Figure 14.

## 4. Discussion 

In this study, seven new molecules were designed to interact with selected COVID-19 proteins. The interaction of molecules with target NSPs and their binding ability have been demonstrated by molecular docking studies. According to results, both ZG-series compounds (1-7) and selected model compounds (hydroxychloroquine and remdesivir) generally bind to the NSP12-RNA complex with higher affinity than the NSP12 single protein and other selected target proteins. 

Sulphur is very nucleophilic due to its large size which makes it easily polarizable. The S-atom in compounds ZG-2 and ZG-7 demonstrates H-bond interactions with the catalytic residue Asp760 of NSP12, and the S-element in ZG-4 and ZG-5 with the catalytic residue Asp623 of NSP12. The S-atom in ZG-3 and ZG-4 can form only one H-bond with the Uracil (pU20) of primer RNA bond to NSP12 protein. Generally, N-groups in aliphatic and cyclic structures in ZG-series compounds form strong and dense H-bonds with critical catalytic residues of NSP12 protein. All these molecular interaction properties enhance the NSP12 inhibition potential of the proposed ZG-series compounds. All proposed compounds have high docking scores comparable to existing antiviral drugs that are currently being used.

In order to prove stable interaction between NSP12 and proposed compounds, molecular dynamic simulation was performed. ZG 7 has been chosen as model compound. According to the results of RMSD plot and the protein-compound poses at different time frames, it was showed that ZG 7 compound interacts stably with the NSP12 protein. 

Two molecules, ZG-5 and ZG-7, which showed the highest activity were selected and synthesized by one-pot reaction at room temperature with acceptable yields. The characterization of the compounds prove that the suggested molecules can be easily synthesized by this method. The molecular design allows to increase activity by making various changes on molecules. Since the synthesis method does not require harsh conditions, the starting materials are commercially available, and the obtained yields are acceptable, the synthesis of the proposed drugs can be carried out quickly and economically. All these advantages show that the molecules will be the new class of drugs that can be used in the treatment of COVID-19.
